# Chemotherapy prescribing errors: an observational study on the role of information technology and computerized physician order entry systems

**DOI:** 10.1186/1472-6963-13-522

**Published:** 2013-12-17

**Authors:** Marianna Aita, Ornella Belvedere, Elisa De Carlo, Laura Deroma, Federica De Pauli, Lorena Gurrieri, Angela Denaro, Loris Zanier, Gianpiero Fasola

**Affiliations:** 1 Department of Oncology, S. Maria della Misericordia, University Hospital, Udine, Italy; 2 Department of Oncology, York Teaching Hospital, York, UK; 3 Regional Coordinator Centre for Rare Diseases, University Hospital of Udine, Udine, Italy; 4 Department of Medical Oncology, University Hospital of Trieste, Trieste, Italy; 5 Health Directorate, Friuli Venezia-Giulia Region, Trieste, Italy

**Keywords:** Adverse drug event, Chemotherapy, Computerized physician order entry (CPOE) systems, Information technology, Prescribing errors, Outpatient

## Abstract

**Background:**

Chemotherapy administration is a high-risk process. Aim of this study was to evaluate the frequency, type, preventability, as well as potential and actual severity of outpatient chemotherapy prescribing errors in an Oncology Department where electronic prescribing is used.

**Methods:**

Up to three electronic prescriptions per patient record were selected from the clinical records of consecutive patients who received cytotoxic chemotherapy between January 2007 and December 2008. Wrong prescriptions were classified as incomplete, incorrect or inappropriate. Error preventability was classified using a four-point scale. Severity was defined according to the Healthcare Failure Mode and Effect Analysis Severity Scale.

**Results:**

Eight hundred and thirty-five prescriptions were eligible. The overall error rate was 20%. Excluding systematic errors (i.e. errors due to an initially faulty implementation of chemotherapy protocols into computerized dictionaries) from the analysis, the error rate decreased to 8%. Incomplete prescriptions were the majority. Most errors were deemed definitely preventable. According to error presumptive potential for damage, 72% were classified as minor; only 3% had the potential to produce major or catastrophic injury. Sixty-eight percent were classified as near misses; adverse drug events had no or little effect on clinical outcome.

**Conclusions:**

Chemotherapy prescribing errors may arise even using electronic prescribing. Although periodic audits may be useful to detect common errors and guide corrective actions, it is crucial to get the computerized physician order entry system and set-ups correct before implementation.

## Background

Chemotherapy administration is an error-prone, high-risk process [1,2]. The reasons are well known. The number and complexity of chemotherapy regimens are increasing steadily; most cytotoxic drugs have a narrow therapeutic range; dose adjustments are often needed; cancer patients are particularly susceptible to drug interactions [3]; and medication delivery is an intricate process, with each step being a potentially significant source of error.

Computerized physician order entry (CPOE) systems are widely regarded as being crucial to reduce hospital medication errors. In the American Society of Health-System Pharmacists (ASHP) 2002 guidelines on preventing chemotherapy prescribing errors, CPOE systems are claimed to offer superior results over pre-printed prescription forms, due to additional features such as the removal of interpretation/transcription errors, the availability of information about drug doses/schedules, the automatic calculation of medication doses, as well as alert and error-checking functions [4].

Relatively few studies have investigated the impact of computerized systems on the reduction of medication errors in oncology; most evidence comes from other specialties or is derived from data about the use of standardized paper prescription forms. Moreover, available data are often conflicting. A meta-analysis of 12 studies showed a 66% overall reduction (odds ratio = 0.34; 95% confidence interval 0.22-0.52) in medication errors when a CPOE system was employed [5]. On the other hand, a qualitative study identified 22 types of medication error risks facilitated by the use of a CPOE system [6]. Examples included patient or medication selection errors due to fragmented CPOE displays preventing a coherent view of patients’ details and medications, pharmacy inventory displays mistaken for dosage guidelines, ignored antibiotic renewal notices placed on paper charts rather than in the CPOE system, medication discontinuation failures, immediate orders and *pro re nata* (PRN) medication discontinuation faults, double dosing and incompatible orders facilitated by separation of functions, and wrong orders due to inflexible ordering formats. Indeed, the variability of error definitions and classification systems among different studies makes the interpretation and comparison of results very difficult. Overall, little is known on the type and frequency of prescribing errors in cancer patients and there is no conclusive evidence that information technology (IT) may exert any specific influence over them.

The main aim of the present study was to evaluate the frequency of chemotherapy prescribing errors in an Oncology outpatient unit equipped with a CPOE system. A secondary aim was to stratify these errors by type, preventability, potential severity as well as actual clinical impact. The study was part of the Italian National Health Service project “Management of cancer patients: procedures for good clinical practice and risk management supported by information systems”.

## Methods

### Setting

This retrospective, observational study was conducted at the Department of Oncology, University Hospital of Udine, Italy. The local information system, named G2, is entirely home-grown and was first introduced in 2001. It initially consisted of a core element, i.e. a CPOE program for chemotherapy prescription, which was used exclusively by the Department of Oncology. Over the years, G2 was gradually developed into an electronic medical record system covering all the aspects of patient care and it is now used by all the Departments at Udine University Hospital and several other Hospitals in North-East Italy. The system allows physicians to manage, store and retrieve all relevant patient information, to schedule outpatient appointments and treatments, to prescribe therapies, and to draw-up clinical letters and discharge summaries. In particular, the G2 CPOE system offers several features potentially improving the safety and efficiency of chemotherapy prescribing, such as an up-to-date glossary of chemotherapy drugs and regimens, the computerized calculation of drug doses (based on anthropometric and/or biochemical variables), recommendations for dose adjustments, patient take-home instructions, and some alert functions (e.g. warning messages in case of overdosage risk or when critical values for the correct calculation of drug doses are missing).

### Eligibility criteria and record screening

Computerized prescriptions were selected from medical records of consecutive cancer outpatients who received active treatment between 1st January 2007 and 31st December 2008 at the Oncology Department in Udine. At the study start date, the implementation of the G2 CPOE system had been successfully completed and the system had reached a stage of full operation.

Prescriptions for cytotoxic chemotherapy regimens were eligible. Exclusion criteria were: (i) prescriptions issued by not fully qualified oncologists, i.e. supervised oncology trainees; (ii) prescriptions for treatments within a clinical trial. A maximum of three prescriptions for each record were analyzed. Prescriptions were independently reviewed by two medical oncology residents specifically trained for this purpose. Both the online prescription and its printed version were considered; in case of inconsistency between the two versions, the latter was deemed to prevail, since the prescription hard copy still represents the basis document for chemotherapy preparation. Thus, if an error in the electronic prescription had been corrected in the printed version, that prescription was considered correct and the error was not taken into account in the analysis.

A specifically developed form was used to register essential demographic and clinical data of study patients and, where appropriate, the error description and its categorization by type, potential preventability, potential severity and actual clinical impact. Data were collected in pseudo-anonymized form.

Two medical oncology specialists independently reviewed all compiled forms for completeness, accuracy and correctness. Disagreements were solved by discussion until consensus was reached. In the event of persistent disagreement, the study coordinator was competent.

The study was approved by the Independent Ethics Committee of the Udine University Hospital, Udine, Italy.

### Error classification and analysis

Chemotherapy prescribing errors were both evaluated as a whole and classified by error type, potential preventability, potential severity and actual clinical impact.

Error types were identified adapting the classification system proposed by Potts and colleagues [7] (Table 1). The appropriateness of prescriptions was evaluated according to the drug registration trials, label information and national/international guidelines [8-10]. As for correctness, all dosages were verified using a web-available instrument for the automatic calculation of the body surface area (DuBois Formula) [11] and a carboplatin AUC calculator based on the modified Cockcroft-Gault method [12]. Possible drug interactions were searched for using a free Drug Interaction Checker software [13]; only interactions with a potentially major level of severity were considered as “errors”.

**Table 1:** **Definition of wrong prescription by error type **[7]

***Categories***	***Definition***
Inappropriate prescription	No support for that regimen at such doses, in that setting or patient, according to age, performance status, baseline organ function, comorbidities, tumor type/stage, potential for drug interactions/allergic reactions, etc.
Incomplete prescription	Missing dosage, unit of measure, administration route, type and volume of infusion solutions, infusion time, premedication drugs, etc.
Incorrect prescription	Medication order showing wrong drug, wrong dosage (depending on variations of the body surface area, organ function, or previous toxicities), wrong unit of measure, wrong administration route, wrong type and volume of infusion solutions, wrong infusion time, etc.

We assessed potential preventability of errors adapting a validated Likert scale to differentiate between definitely not preventable, probably not preventable, probably preventable, and definitely preventable errors [14] (Table 2).

**Table 2:** Definition of potential preventability, potential severity and actual clinical impact

	***Categories***	***Definition***
Potential preventability [14]	Definitely preventable	NS^a^
	Probably preventable	NS^a^
	Probably not preventable	NS^a^
	Definitely not preventable	NS^a^
Potential severity [15]	Minor	No injury, nor increased length of stay nor increased level of care.
	Moderate	Increased length of stay or increased level of care for 1 or 2 patients.
	Major	Permanent lessening of bodily functioning, disfigurement, surgical intervention required, increased length of stay for 3 or more patients, increased level of care for 3 or more patients.
	Catastrophic	Death or major permanent loss of function, suicide, rape, hemolytic transfusion reaction. Surgery/procedure on the wrong patient or wrong body part, infant abduction or infant discharge to the wrong family.
Actual clinical impact [16,17]	Preventable ADE^b^	Injuries resulting from an error at any stage throughout the medication process.
	Near misses	Errors detected and intercepted before any harm is done.

^a^NS: not specified; ^b^ADE: Adverse Drug Event.

Error potential severity, based on the plausible level of patient injury, was classified using the Healthcare Failure Mode and Effect Analysis (HFMEA) Severity Rating Scale [15] (Table 2). To assess their real clinical impact, errors were distinguished among near misses [16] and preventable adverse drug events (ADE) [17] (Table 2). The severity of ADE was judged using the same rating scale as above [15].

The analysis of all the secondary endpoints (type of error, degree of preventability, severity and clinical impact) was pre-planned.

## Results

### Prescription selection

**Figure 1:**
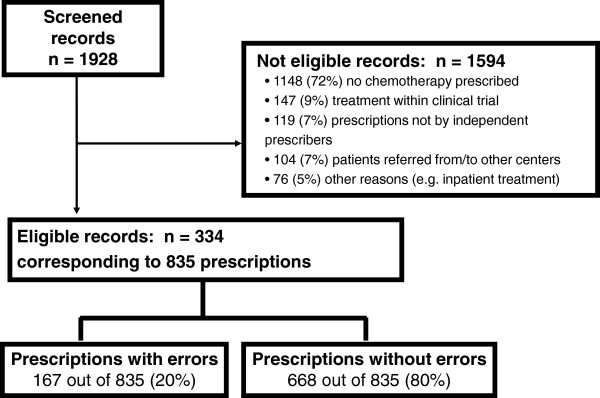
Schematic representation of the study results.

Medical records of 1928 patients who attended the Outpatient Oncology Department at the Udine University Hospital between 1st January 2007 and 31st December 2008 were screened (Figure 1). Eighty-three percent (1594 out of 1928) of screened records were not eligible; main reasons for exclusion are shown in Figure 1.

Overall, 334 records were eligible; up to three prescriptions for each eligible record were selected, giving a total of 835 prescriptions (Figure 1). One, two and three prescriptions were analyzed in 55, 57 and 222 records, respectively.

Patient median age was 64 years (interquartile range: 54–70); 61% of patients were females. Breast, colorectal, other gastrointestinal tumors and lung cancer were the most common tumor types (33%, 23%, 13% and 12% of all eligible cases, respectively). Most patients had locally advanced (52%) or metastatic (32%) disease.

### Error analysis

Records containing one or more prescribing errors were 79 out of 334 (24%). Prescriptions with errors were 167 out of 835, for an overall error rate of 20%. Eleven prescriptions (1%) contained more than 1 error, for an overall number of 181 errors (Figure 1).

Incomplete prescriptions were the most frequent (66%), followed by incorrect and inappropriate prescriptions (28% and 6%, respectively). Incomplete prescriptions (n = 110) failed to specify the administration route (n = 101), the infusion solution volume (n = 2) or the number of capsules to be dispensed in case of oral chemotherapy (n = 7). Incorrect prescriptions (n = 47) included: errors of over/underdosage, due to failure in modifying dose based on previous toxicity or on variations of biochemical parameters; and re-exposure to drugs causing a previous reaction. Inappropriate prescriptions (n = 10) included: errors in drug dose(s) or treatment selection with respect to patient age, tumor type or organ function (n = 8); omission of primary prophylaxis with a granulocyte colony-stimulating factor in a patient at high risk of febrile neutropenia (n = 1); prescription in the absence of recent full blood count and biochemistry results (n = 1).

The analysis by error type revealed a significant proportion of systematic errors which were derived from errors in the predefined chemotherapy protocols, such as failure to specify route of administration or volume of infusion solution; these errors were often present in prescriptions which would have been otherwise error-free. We labeled these systematic errors as *a priori* errors. Wrong prescriptions due to *a priori* errors were 103. After excluding these errors, the overall error rate declined from 20% to 8%. The proportion of different error types also changed substantially: incomplete prescriptions rate fell from 66% to 11%, whereas the rate of incorrect and inappropriate prescriptions raised from 28% to 73% and 6% to 16%, respectively.

The majority of errors were deemed probably (33%) or definitely (66%) preventable.

An effort was made to judge error severity taking in consideration their presumptive potential for damage, independently from clinical outcomes. In this perspective, 72% and 25% of errors were classified as minor and moderate, respectively, whereas 2% and 1% had at least the potential to produce major or catastrophic injuries.

With regards to the actual clinical consequences of the identified errors, sixty-eight percent were classified as near misses. Registered ADE had no or little effect on patient clinical outcome; there were no major or catastrophic ADE. The potential severity, potential preventability, and actual clinical impact of the identified errors are summarized in Table 3.

**Table 3:** Breakdown of errors by potential severity, preventability and actual clinical impact

	**All errors n = 181**
Potential severity, n (%)	
Minor	130 (72)
Moderate	45 (25)
Major	4 (2)
Catastrophic	2 (1)
Potential preventability, n (%)	
Definitely preventable	120 (66)
Probably preventable	60 (33)
Probably not preventable	1 (1)
Not preventable	0 (0)
Actual clinical impact, n (%)	
Preventable ADE^a^	58 (32)
Near misses	123 (68)

^a^ADE: Adverse Drug Event.

## Discussion

Medication errors remain one of the most common causes of morbidity and death among patients [18]. While the medication process consists of three phases, namely prescribing, dispensing and administration, most of the medication errors occur during the first phase [13,19-22]. Prescribing errors may have catastrophic consequences, especially when involved drugs have narrow therapeutic index, as it is the case of chemotherapy. The use of IT has been advocated to reduce the occurrence of medication errors [4]. Specifically, CPOE systems may reduce the frequency of prescribing and probably dispensing errors, whereas other IT solutions (e.g. barcode assisted medication administration and radio frequency identification technologies) may reduce the occurrence of dispensing and administration errors.

The potential for errors, however, remains even using IT. In our study, set in an outpatient Oncology unit where a CPOE system is in use, we observed an overall chemotherapy prescribing error rate of 20%, which is higher than previously published reports from oncology settings [23-31]. Possible explanations for such a difference include the use of different CPOE systems, variability in the definition of prescribing errors and also differences in study design and error calculation. In our study, most of the errors were actually due to mistakes in the configuration of system information, which were automatically incorporated into otherwise error-free prescriptions; we have labeled these system-induced prescribing errors as “*a priori* errors”. Excluding the *a priori* errors, error frequency fell to 8%, a figure similar to those reported in previous studies [23,29]. Most important, virtually all these *a priori* errors were formal rather than substantial errors.

The high proportion of system-induced errors highlights the importance of standardization of computer systems, which creates a uniform model that may reduce the complexity and variability of a specific process [32]. Standardization may be a potential source of systematic error. In our study we found situations in which the CPOE system not only introduced *a priori* errors but also facilitated *de novo* errors. Most of them were related to “Copy and Paste” functions. When used right, these functions are of unquestionable value. However, they may also raise several problems [33]. Probably, the most dangerous occurrence is that of a medical prescription created with a simple order (“generate a new cycle”) as a copy of a previous prescription, with no need to confirm relevant information (patient weight, organ function, previous toxicity requiring dose reduction). Among 47 incorrect prescriptions in our study, most consisted in errors of over/underdosage, due to failure in modifying the dose based on toxicity or on variations of biochemical parameters after the new cycle prescription had been generated as a copy of the previous cycle. One could argue that operators should always maintain an “attentional control mode” to monitor automatic functions [33]. Since in our system a printed copy is always associated with the electronic prescription, physicians would have had the possibility of manually editing faulty prescriptions. Unfortunately, using the attentional mode is “effortful and difficult to sustain for more than brief periods” [34]. For this reason, stakeholders have already noted that it is vital to get protocols right the first time, as their complexity often makes it difficult to detect configuration errors later on. In some realities a dedicated human resource has been assigned to constantly refine the protocols based on feedback from clinicians and to develop new pathways for the management of new medicines [35]. As a result of this study, we have corrected all the mistakes identified in the CPOE system settings, i.e. mistakes in the dictionaries of the predefined chemotherapy protocols and we have undertaken an in-depth review of existing protocols and dictionaries to check their accuracy. Currently, we are working on an updated version of the CPOE system, in which all information functions are separated in two domains: the first is “a context area”, with clinical data on patients’ history, past and current treatments, laboratory/radiological exams; in the second section, alerts will play a key role in the management of compelling information, as the system will detect the lack of crucial data and hold up the access to critical functions until the appropriate field is filled in.

The errors identified in this study had no or little impact on patients. Briefly, the majority of errors (68%) were near misses. These errors were intercepted by a pharmacist, chemotherapy nurse or clinician, and corrected before any harm was caused to the patient. Specifically, most of these errors were systematic prescribing errors due to errors in the predefined chemotherapy protocols (the *a priori* errors); examples of these errors are failure to specify the administration route, solution volume or infusion time in the prescription. In these cases, the errors were detected and the prescriptions amended before chemotherapy was prepared and/or administered. The remaining errors were not detected and resulted in ADE. These events, all potentially preventable, did not result in significant harm to the patient. Examples are errors of chemotherapy dose due to failure in recalculating the chemotherapy dose based on up-to-date body weight or taking into account changes in organ function or previous toxicity, omission of adequate allergy prophylaxis following a reaction as per local guidelines, omission to prescribe prophylactic G-CSF in a patient at high risk of neutropenic fever. None of these patients experienced significant toxicity as a result of the error but we acknowledge that these errors had the potential for major or catastrophic consequences in a few cases. Of note, most of these errors are prevented in the updated version of the CPOE system with the presence of mandatory fields for up-to-date weight, critical blood results and alerts in case of inadequate parameters/values.

It has been suggested that comparing the frequency of medication errors among different healthcare organizations is meaningless, due to differences in culture, definition of medication error, patient populations served, and types of detection and reporting systems [36]. While we agree that some differences - such as the particular composition of patient population, or the system used to identify errors and adverse events - may be difficult to overcome, we firmly believe that reporting of events is worthwhile, provided that data are analyzed and that analysis stems from a shared classification*.* Indeed, such an approach might start the process of developing generalized solutions. Unfortunately, a number of systems have been used so far, and taxonomic differences make data difficult to be shared*.* No classification system has been validated, nor has demonstrated to offer significant improvements in patient safety. A major limitation of our research seems to lie in the degree of classification detail: even when we simply considered the potential for damage of identified errors - rather than their real outcome - more than 90% were judged to be minor/moderate errors. There is a need for a more detailed and standardized definition and classification of prescribing errors, to collect useful and usable information which may ultimately be applied to improve patient care, guide health policy planning and perform good quality research [37].

Several other study limitations should be acknowledged, mainly the level of training and endeavor needed to identify errors through charts review and data collection; the lack of standardization of this error-capturing approach; and the weakness and possible biases of a quality observational study.

## Conclusions

The present study provides further evidence that chemotherapy prescribing errors arise even when CPOE systems are used. It is crucial that protocols are set up correctly before implementing electronic prescribing. Once the CPOE goes live, periodic audits are needed to detect and promptly correct common errors related not only with the existing protocols but also with the new chemotherapy protocols constantly added to the CPOE dictionaries.

Additional evaluation of CPOE systems for chemotherapy prescribing is needed and should be the focus of large prospective and ideally multicenter studies. The development of standard definitions for error type and the use of robust study designs are vital.

## Abbreviations

CPOE: Computerized physician order entry; IT: Information technology; ASHP: American Society of Health-System Pharmacists; HFMEA: Healthcare failure mode and effect analysis; ADE: Adverse drug event.

## Competing interests

The authors declare that they have no competing interests.

## Authors’ contributions

Concept and design: MA, OB, LD, GF; data collection and assembly: all authors; data analysis and interpretation: MA, OB, LD, GF; manuscript writing: MA, OB, GF. All authors read and approved the final manuscript.

## Pre-publication history

The pre-publication history for this paper can be accessed here:

http://www.biomedcentral.com/1472-6963/13/522/prepub
